# Genetic characterization of an insect-specific flavivirus isolated from *Culex theileri* mosquitoes collected in southern Portugal

**DOI:** 10.1016/j.virusres.2012.04.010

**Published:** 2012-08

**Authors:** Ricardo Parreira, Shelley Cook, Ângela Lopes, António Pedro de Matos, António Paulo Gouveia de Almeida, João Piedade, Aida Esteves

**Affiliations:** a Unidade de Microbiologia Médica, Grupo de Virologia, Instituto de Higiene e Medicina Tropical (IHMT), Universidade Nova de Lisboa (UNL), Rua da Junqueira 100, 1349-008 Lisboa, Portugal; b Unidade de Parasitologia e Microbiologia Médicas (UPMM), IHMT/UNL, Rua da Junqueira 100, 1349-008 Lisboa, 10 Portugal; c Natural History Museum, Cromwell Road, London SW7 5BD, UK; d Serviço de Anatomia Patológica, Hospital Curry Cabral, Rua da Beneficência 8, 1069-166 Lisboa, Portugal; e Unidade de Parasitologia Médica, IHMT/UNL, Rua da Junqueira 100, 1349-008 Lisboa, Portugal

**Keywords:** Insect-specific flavivirus, Phylogenetic analysis, Iberian Peninsula, Portugal, Complete genomic sequence

## Abstract

We describe the full genetic characterization of an insect-specific flavivirus (ISF) from *Culex theileri* (Theobald) mosquitoes collected in Portugal. This represents the first isolation and full characterization of an ISF from Portuguese mosquitoes. The virus, designated CTFV, for *Culex theileri* flavivirus, was isolated in the C6/36 *Stegomyia albopicta* (=*Aedes albopictus*) cell line, and failed to replicate in vertebrate (Vero) cells in common with other ISFs. The CTFV genome encodes a single polyprotein with 3357 residues showing all the features expected for those of flaviviruses. Phylogenetic analyses based on all ISF sequences available to date, place CTFV among *Culex*-associated flaviviruses, grouping with recently published NS5 partial sequences documented from mosquitoes collected in the Iberian Peninsula, and with Quang Binh virus (isolated in Vietnam) as a close relative. No CTFV sequences were found integrated in their host's genome using a range of specific PCR primers designed to the prM/E, NS3, and NS5 region.

## Highlights

► A new insect flavivirus (CTFV) was isolated from *Culex theileri* mosquitoes. ► CTFV does not replicate in Vero cells. ► Phylogenetic analyses place CTFV among *Culex*-associated flaviviruses. ► CTFV seems to be dispersed in the Iberian Peninsula. ► CTFV sequences were not found in theirs hosts’ genome.

## 1 Introduction

The genus *Flavivirus* (*Flaviviridae*) includes over seven dozen known enveloped viruses with ssRNA (+) genomes encoding a single polyprotein which is co-translationally processed by viral and host proteases into structural, and non-structural proteins, that play critical roles in viral replication (Lindenbach and Rice, 2003). Interest in the flaviviruses has been mostly fueled by their impact on human and animal health. Furthermore, they also represent useful models for evolutionary analysis of the transmission modes of vector-borne viruses, as the genus includes viruses vectored by mosquitoes (e.g. Dengue Fever virus (DENV), Yellow Fever virus (YFV), West Nile virus (WNV), and the Japanese Encephalitis virus (JEV)), ticks [e.g. Tick-Borne Encephalitis virus (TBEV)], and those with no known vector (NKV) viruses (Cook and Holmes, 2006).

Phylogenetic trees of the flaviviruses tend to suggest three main monophyletic clusters. One includes mosquito-borne viruses, a second contains tick-borne viral agents, while a third comprises viruses with no known invertebrate host (NKV). Although some NKV viruses do segregate within the mosquito-borne clade, this seems to reflect the secondary loss of transmission by an arthropod (Cook and Holmes, 2006, Gould et al., 2003). Over time, a fourth tentative group of flaviviruses has been discovered, containing strains which appear to have no known vertebrate host and which, in earlier analyses, have been used to root phylogenetic trees of the genus. The recent identification of viral sequences related to flaviviruses integrated in the genome of mosquitoes, further complicates the evolutionary history of this group of viral agents (Crochu et al., 2004, Roiz et al., 2009).

The analysis of a large number of mosquitoes in natural populations indicates that these viruses form a genetically diverse group with a wider geographical dispersion, and prevalence, than initially suspected (Blitvich et al., 2009, Crabtree et al., 2003, Cook et al., 2006, Farfan-Ale et al., 2009, Hoshino et al., 2007, Hoshino et al., 2009, Morales-Betoulle et al., 2008). Their inclusion in the genus *Flavivirus* is supported by similarities to accepted flaviviruses in terms of genomic organization, polyprotein hydropathy profiles and cleavage sites, but unlike most flaviviruses, they do not seem to replicate in vertebrate cells, at least *in vitro* (Cammisa-Parks et al., 1992, Crabtree et al., 2009, Hoshino et al., 2007, Hoshino et al., 2009, Kuno, 2007), justifying their designation as “insect-specific flaviviruses” (ISF) (Cook et al., 2006, Cook et al., 2009, Cook et al., 2011, Farfan-Ale et al., 2009).

In Portugal, and especially in the summer, *Cx. theileri* (Theobald), is found in high densities in field collections using CO_2_-baited CDC traps, especially when carried out in the coastal, and estuarine, districts of Setúbal and Faro (Almeida et al., 2008, Almeida et al., 2010). During the summers of 2009 and 2010, we carried out a large entomological survey in the south of Portugal, which resulted in the collection of over 36,000 adult mosquitoes, in which a large prevalence of flavivirus-specific sequences was detected in different species (Costa et al., 2011). In this study we report the isolation of four ISF strains from pools of *Culex theileri*. Herein we present the genetic characterization and analysis of near full-length genomic sequences of two viral strains, tentatively designated CTFV (for *Culex theileri* flavivirus), including their phylogenetic relationships with other flaviviruses.

## 2 Results

### 2.1 Viral isolation and preliminary characterization

Sequences related to flavivirus NS5 coding regions (RNA-dependent RNA polymerase) were initially detected by nested RT-PCR (Flavi1*/Flavi2* primers in a first round, followed by Flavi3*/Flavi2* primers in the second; see supplementary Table 1), using RNA extracted from 4 macerates (laboratory code number 132, 153, 178, and 210) prepared from pools of mosquitoes identified morphologically as *Cx. theileri* (see Section 4). To further confirm these identifications, DNA was extracted from the flavivirus-positive mosquito pool, and part of the region coding for the “barcoding” section of the mitochondrial COI gene (mitochondrial cytochrome c oxidase) was amplified (supplementary Table 1), cloned (see Section 4) and a total of 4 individual clones were sequenced. These COI sequences were compared with those present in the BLAST database (including sequences obtained from voucher specimens identified and accessioned into the Collections at the Natural History Museum), and a 100% identity was found to those from *Cx. theileri* voucher mosquitoes. Species identification based on BOLD-IDS (supplementary data 1) also confirmed both the BLAST results and the initial taxonomic assignments based on morphology.

**Fig. 1:**
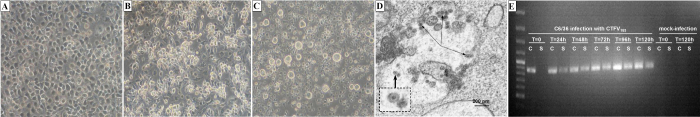
Microscopic observation of C6/36 cells: mock-infected cells (A; 400×), or after infection (day 3) with CTFV strain 153 (B; 400×) or CFAV (C; 200×). (D) Transmission electron micrograph of a thin section of C6/36 cells infected (day 2 post-infection) with CTFV strain 153 (thin arrows) showing multiple round, enveloped viral particles with an electron dense core (thick arrow) accumulated in enlarged cytoplasmic vesicles (scale bar, 200 nm). (E) Kinetics of CTFV_153_ RNA detection in C6/36 infected and mock-infected cells. At different times after infection, total RNA was extracted from the culture supernatant (S) and cell-sediment (C). After reverse-transcription, a virus specific fragment was amplified with primers SeqC and SeqD (supplementary Table 1). The GeneRuler 1 kb Plus DNA ladder (Fermentas, Vilnius, Lithuania) was used as a molecular weight marker.

For viral isolation, filter sterilized aliquots of mosquito macerates were used to inoculate monolayers of C6/36 cells, which were then observed for cytopathic effect (CPE). After the third weekly blind passage, and when compared to the negative controls (Fig. 1A), CPE characterized by cell growth retardation and the formation of cellular aggregates was seen in the cultures inoculated with *Cx. theileri* macerates (132, 153, 178 and 210) (Fig. 1B). This was different from the CPE seen in C6/36 cell cultures infected with the CFAV, which typically exhibit differently sized syncytia (Fig. 1C). No CPE was observed in Vero cell cultures inoculated with an aliquot of infected C6/36 supernatant after three blind passages (not shown).

No amplification products were observed if RNA extracted from CTFV-infected C6/36 culture supernatants was directly used as amplification template without prior cDNA synthesis (data not shown). However, specific amplicons were obtained when the RT-PCR proceeded to completion, indicating that the CTFV genome is an RNA molecule.

Virus particles, with morphology compatible with that of flaviviruses, were visualized by electron microscopy of C6/36 cells at 48 h post-infection. Virions displayed an approximate diameter of 50 nm and a dense core (Fig. 1D) surrounded by an envelope (not shown), and were frequently observed in association with the enlarged membrane-bound cisternae, being absent from mock-infected cells.

Viral replication in C6/36 cells appeared to be rapid, as viral RNA could be detected in culture supernatants 24 h after infection with CTFV_153_ (Fig. 1E), while, immediately after infection, viral RNA could only be detected in the cell sediment (incoming viruses).

### 2.2 Amplification of the near full-length genomes of CTFV

The analysis of a small NS5-specific sequence fragment (see above) revealed over 90% identity (BLASTn) with two very short (∼200 bp) sequences amplified from *Cx. theileri* (EU716420) and *Cx. fuscocephala* (Theobald) (AY457040), and over 88% identity with the corresponding NS5 sequences of several putative flaviviruses isolated from different mosquito species via BLASTx. A preliminary phylogenetic tree, constructed with these sequences, indicated their inclusion in a monophyletic cluster along with ISFs isolated from different sources and geographic regions. CTFV and the sequences represented by EU716420 and AY457040 form a robust monophyletic clade (supplementary data 2). However, due to the small size of the analyzed amplicon, a larger section (1.3 kb) of the viral NS5 gene was obtained by PCR using the F4/Flavi2* primers (supplementary Table 1). These amplicons were cloned and sequenced (1 for isolates 132, 178 and 210, and two for the 153 strain), and their phylogenetic analysis (NJ-tree; supplementary data 3) showed that the CTFV formed a monophyletic cluster, supported by maximum bootstrap values, and characterized by low genetic diversity. This was included in a larger assemblage of ISF sequences which segregated away from all mosquito-, tick- or NKV-flaviviruses (100% bootstrap).

Given its similarity with other *Culex*-specific flaviviruses, a multiple sequence alignment was constructed including the full-length (or near full-length) genomes of seven *Culex*-associated viral sequences downloaded from public databases (see Section 4), which enabled the design of several PCR primers complementary to sequences scattered across the whole of the viral genome (supplementary Table 1) and allowing the amplification of the near full-length genomes of two viral strains (153 and 178 were randomly selected), isolated from pools of mosquitoes collected in 2009 and 2010, approximately 300 km apart (districts of Faro and Setúbal).

Since the amplification of CTFV sequences was exclusively based on PCR using primers targeting conserved regions in the viral genome, in both cases (CTFV_153_ and CTFV_178_) the 5′- and 3′-UTR (untranslated regions) sequences obtained were incomplete. Their sizes were restricted to 50 nt (5′-UTR) and 416 nt (3′-UTR) in length, which correspond, respectively, to about 50% and 63% of the expected size, taking into account what is reported for different *Culex*-associated ISF (≈100 nt/5′-UTR, ≈660 nt/3′-UTR). Both viral genomes, differing in only 1.1% of their nt sequence, include a single open reading frame (ORF) encoding a putative polyprotein with 3,357 amino acid residues.

No amplification of CTFV sequences by RT-PCR was achieved using the F4/Flavi2* (NS5-specific amplicon), SeqC/D (NS3-specific amplicon), or SeqF/G (prM/E-specific amplicon) pairs of primers and RNA extracted from the supernatants of Vero cells inoculated with CTFV_153_ or CTFV_178_. These results, in combination with the absence of CPE in Vero cells (see above), suggest that CTFV does not replicate in this cell line, which is susceptible to most arthropod-borne viruses. Similarly, no amplification was obtained using the these same pairs of primers and genomic DNA extracted from either Vero or C6/36 cultures infected with CTFV or from any of the mosquito macerates used, with no reverse transcription step (data not shown).

### 2.3 Phylogenetic analysis of CTFV sequences

**Fig. 2:**
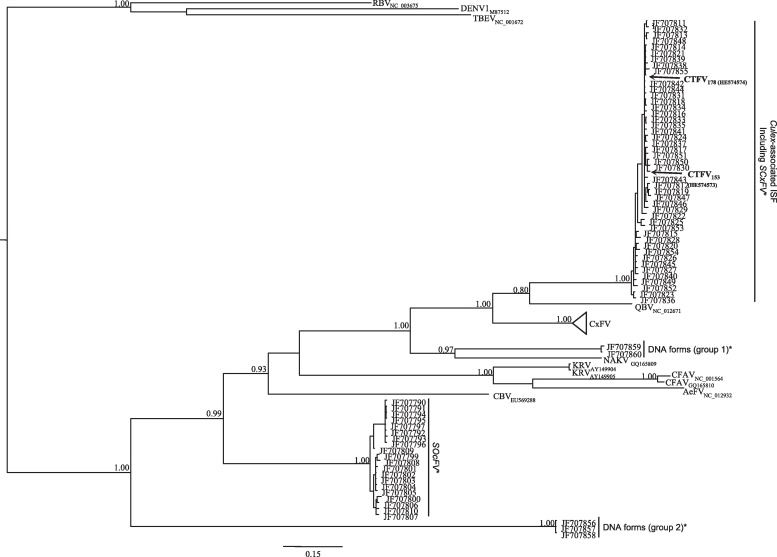
Bayesian phylogenetic analysis of partial flavivirus NS5 nucleotide sequences. Posterior probability values ≥0.80 are indicated at specific branches. The sequences used are denoted by viral name (*Aedes* flavivirus – AeFV; Calbertado virus – CBV; cell fusing agent virus – CFAV; *Culex* flavivirus – CxFV; *Culex theileri* flavivirus – CTFV; Kamiti River virus – KRV; Nakiwogo virus – NAKV; Quang Binh virus – QBV; Tick-Borne Encephalitis virus – TBEV; Rio Bravo virus – RBV; Dengue virus serotype 1 – DENV1), and accession number. The sequences solely indicated by their accession number and referred with an asterisk [*SCxFV* (Spanish *Culex* flavivirus), *SOcFV* (Spanish *Ochlerotatus* flavivirus), DNA forms (group 1), and DNA forms (group 2); “DNA forms” indicate these sequences were directly obtained from the amplification of mosquito DNA] were those described by Vázquez et al. (2012). The CTFV sequences here reported are highlighted in bold-face. The size bar indicates 15% of genetic distance.

**Fig. 3:**
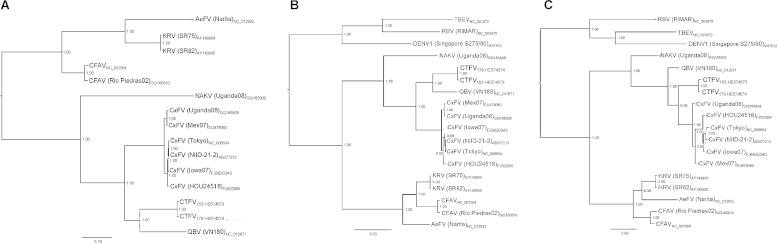
Bayesian phylogenetic analysis of flavivirus ORF (A), NS3 (B) and NS5 (C) amino acid sequences. Posterior probability values ≥0.95 are indicated at specific branches. The sequences used are denoted by viral name (*Aedes* flavivirus – AeFV; cell fusing agent virus – CFAV; *Culex* flavivirus – CxFV; *Culex theileri* flavivirus – CTFV; Kamiti River virus – KRV; Nakiwogo virus – NAKV; Quang Binh virus – QBV; Tick-Borne Encephalitis virus – TBEV; Rio Bravo virus – RBV; Dengue virus serotype 1 – DENV1), viral strain (in parentheses) and accession number. The size bars indicate 10% (A), 20% (B), and 9% (C) of genetic distance.

A full analysis of the phylogenetic relationships between CTFV and other flaviviruses was carried out using alignments of (i) near full-length nt sequences and (ii) the predicted amino acid sequences for the E, NS3, NS5 and ORF CTFV products, following a Bayesian approach (see Section 4). The phylogenetic tree based on alignment of the complete coding sequence (supplementary data 4) resulted in a similar topology to that obtained using in a preliminary analysis of partial NS5 sequences amplified from all the mosquito macerates used (132, 153, 178, and 210) (supplementary data 2 and 3). The two CTFV sequences segregated with the other ISFs in a monophyletic cluster with maximum posterior probability. In particular, the two CTFV strains (153 and 178) formed a monophyletic cluster with sequences isolated from *Culex* mosquitoes, where their closest relative was Quang Binh virus (QBV). Based exclusively on near full-length genome analysis, K2P-corrected genetic distances between CTFV and the QBV (40.3%) would tend to support the assignment of CTFV as a different viral species. However, when the CTFV sequences were compared to the partial NS5 sequences recently published by Vázquez et al. (2012) for both nt (Fig. 2) or amino acid (not shown) datasets, both CTFV strains fell with strong support in a monophyletic cluster of viral NS5 sequences with apparent low genetic diversity, amplified from RNA extracted from both *Cx. theileri* and *Cx. pipiens* (Linnaeus) mosquitoes. Although no complete sequence for these *Culex*-associated viruses was ever reported by Vázquez et al. (2012), our analysis suggests that CTFV (or at least viruses with similar NS5 sequences) circulate widely in the Iberian Peninsula. These viruses are placed in phylogenetic trees as a sister group to QBV. Similar results were obtained based on the phylogenetic analyses of ORF, NS3, NS5 (Fig. 3) or E (supplementary data 5) amino acid sequences.

### 2.4 Analysis of CTFV encoded proteins

The analyses of the CTFV polyprotein hydropathy plots (not shown) with those of different flaviviruses revealed striking similarities in both the structural and non-structural genome sections. Several conserved protein domains, previously recognized in some of its proteolytic products (Table 1), were also detected in the 3357 amino acid CTFV polyprotein (see below). The viral serine-protease seems to be involved in the cleavage of C/anchored C, prM/M, NS2a/NS2b, NS2b/NS3, NS3/NS4a and NS4b/NS5, while a furin-like protease seems to be involved in the processing of the anchored C/prM, M/E, E/NS1 and possibly NS4a/NS4b protein junctions. The proposed cleavage site at the intersection between the NS1 and NS2a proteins could not be assigned to any of the abovementioned proteases.

**Table 1:** Predicted cleavage sites in several flavivirus polyproteins.

Virus	C/AnchC	AnchC/prM	prM/M	M/E	E/NS1	NS1/NS2a	NS2a/NS2b	NS2b/NS3	NS3/NS4a	NS4a/NS4b	NS4b/NS5
**APOIV**_**NC_003676**_	KGG**RR**/**G**GKSV	PIALS/AVVMN	TRT**RR**/DVTIQ	APAYA/STCVS	TGVVG/EIGCM	GLVMA/FDEEP	RSG**QR**/**S**VDPI	RSI**QK**/**S**NTSF	AKG**KR**/**S**GMTI	EGM**QR**/RTQVD	SEN**RR**/**G**VSSS
**ALKV**_**NC_004355**_	RGK**RR**/**S**TTGL	TLVIS/ATIRR	GRS**RR**/**S**VSIP	APTYA/TRCTH	LGVGA/DMGCA	MVLAD/NGAML	RRN**RR**/**S**FSEP	SSG**RR**/**S**ELVF	ASG**RR**/**S**VGDV	PGK**QR**/**S**SDDN	TGT**RR**/**G**GADG
**WNV**_**AJ965628**_	KQK**KR**/**G**GKTG	ASVGA/VTLSN	RHS**RR**/**S**RRSL	APAYS/FNCLG	VNVHA/DTGCA	SQVNA/YNADM	PNR**KR**/**G**WPAT	QYT**KR**/**G**GVLW	ASG**KR**/**S**QIGL	PEK**QR**/**S**QTDN	PGL**KR**/**G**GAKG
**JEV**_**NC_001437**_	KQN**KR**/**G**GNEG	AYAGA/MKLSN	KRS**RR**/**S**VSVQ	APAYS/FNCLG	TNVHA/DTGCA	SQVDA/FNGEM	PNK**KR**/**G**WPAT	KTT**KR**/**G**GVFW	AAG**KR**/**S**AISF	PEK**QR**/**S**QTDN	PSL**KR**/**G**RPGG
**NOUV**_**EU159426**_	VSK**RR**/**G**SASL	GVASA/VTFTT	QRS**RR**/**S**VGIS	IPAYS/MKCIG	TSVSA/ELGCS	LGVLA/MTMMF	KTT**KR**/**S**VPQS	ENR**KR**/**S**NDTP	AGG**KR**/**S**AVDL	EGK**QR**/**S**MVDN	AYK**KR**/**G**IWEV
**KRV**_**NC_005064**_	LEK**QR**/**S**GPNL	GLCYG/EMLRY	VRR**RR**/**A**PQPQ	NVVKA/SSIEP	RSVSA/DVGCG	GKAHA/CSDFR	AAE**R**A/QQPTI	SEQN**R**/**S**DDLL	WDT**RK**/LSIEF	CGVLA/WEMRL	FNQF**R**/**A**LEKS
**LAMV**_**FJ606789**_	KNG**KR**/**S**TEIS	GAAMA/ASMFT	RRG**KR**/**S**VALA	GPAYS/LQCVD	TTTVA/LSEVG	SKVSA/GTFQI	TSG**KR**/**S**WPAG	KSG**RR**/**G**TVLM	AEG**RR**/**S**YVPL	EPGSQ/RSVQD	GVP**RR**/**G**MTIC
**AEFV**_**AB488408**_	LEA**QR**/**S**HSPV	GLALS/ETLRY	PRK**RR**/**S**SPQR	NVVRA/TSIEP	IRRVA/GDIGC	GKADA/TADFH	AAE**R**A/DHPSA	NEHC**R**/**S**DDLL	WDQ**RR**/LSIEM	CSVLA/WEMRL	FSKF**R**/**A**LEKS
**CxFV**_**AB262759**_	LEA**KR**/**S**AKNA	MVLGA/VVIDM	KRE**RR**/VASTN	TTVKG/EFVEP	VYTKA/DVGCG	VTIDA/DGEDM	RAS**RR**/**S**LVAG	VSVF**R**/**S**NEVN	ELD**KR**/**S**KIML	MGVVA/WEMDL	RMAL**R**/**S**LVKT
**QBV**_**NC_012671**_	LEN**RR**/**S**ANPL	TLCGT/MVIDM	KRV**KR**/**A**TEQP	STVKG/EFVEP	YYTRA/DVGCG	LIIEN/EGVEI	LRA**SK**/**R**SALL	TSN**RR**/**S**GVND	ELE**KR**/**S**KIML	MGIVA/WELEL	RLAT**R**/**S**LVKT
**CFAV**_**NC_001564**_	LES**RR**/TTGNP	VLCGC/VVIDM	KRE**KR**/**S**REPP	TTVKG/EFVEP	YYVRA/DLGCG	GKANA/QSDFR	AAE**K**A/HQPTV	TASN**R**/**S**DDLL	WET**RK**/VSIDF	SIGN**R**/**S**YMDS	FNQF**R**/**A**LEKS
**CTFV**	LEN**RR**/**S**ANNP	VLCGC/VIIDM	KRV**KR**/**A**PETP	TTVKG/EFVEP	YFARA/DVGCG	LTIEK/GGQTI	LRAS**K**/**S**SMLL	STAY**R**/**A**GVND	ELE**KR**/TKLSI	MGVVA/WELNL	RGGL**R**/**S**LVKT

The amino acid residues highlighted in bold-face represent cleavage sites for the NS3 serine protease of flaviviruses.

Cleavage sites with underlined residues seem to follow a host signalase cleavage consensus (von Heijne, 1984, Chambers et al., 1990).

AEFV – *Aedes* flavivirus, ALKV – Alkhurma virus, APOIV – Apoi virus, CFAV – cell fusing agent virus, CxFV – *Culex* flavivirus, CTFV – *Culex theileri* flavivirus, JEV – Japanese Encephalitis virus, KRV – Kamiti River virus, LAMV – Lammi virus, NOUV – Nounane virus, QBV – Quang Binh virus, WNV – West Nile virus.

The pairwise sequence identities between the CTFV proteins and those of other flaviviruses fall below 80% (Table 2), the sole exceptions being the NS5 of CTFV and those of CxFV and QBV which share 81.4% and 83.9% of amino acid sequence identity, respectively. The average diversity for the NS5 aligned amino acid sequences of CTFV and Spanish *Culex* flavivirus (SCxFV) reported by Vázquez et al. (2012) was 0.2%, again suggesting that CTFV and SCxFV either correspond to (i) different strains of the same virus or (ii) different viruses coding for almost identical NS5 proteins.

**Table 2:** Comparison of the putative CTFV amino acid sequences with those of other flaviviruses.

Proteins	CTFV HE574573	CxFV AB262759		QBV NC_012671		CFAV NC_001564		WNV AJ965628		JEV NC_001437		YFV NC_002031		TBEV NC_001672		MDV NC_003635	
	Size^a^	Size^a^	ID%	Size^a^	ID%	Size^a^	ID%	Size^a^	ID%	Size^a^	ID%	Size^a^	ID%	Size^a^	ID%	Size^a^	ID%
AnchorC	136	139	44.6	136	49.6	136	52.4	122	17.1	127	16.9	121	16.2	112	23.3	110	18.3
C	115	118	45.8	116	50.9	115	52.5	104	17.7	105	18.2	101	18.3	96	24.8	91	19.0
preM	143	143	75.7	142	76.2	142	74.8	167	18.1	167	17.9	164	18.4	168	18.4	87	11.5
M	59	60	76.7	59	79.7	59	78.0	78	12.3	75	10.6	75	14.7	75	11.5	75	13.2
E	427	427	74.0	427	77.1	422	79.4	501	19.1	500	19.7	493	16.4	496	19.6	482	16.2
NS1	369	369	70.5	369	73.7	395	37.0	352	24.2	352	23.2	352	25.6	352	26.8	353	23.1
NS2a	228	230	62.2	229	63.3	232	18.6	231	14.9	227	16.4	224	14.3	230	16.2	221	13.8
NS2b	143	142	62.9	143	53.1	124	17.1	131	16.6	131	17.7	130	19.2	131	19.0	132	16.7
NS3	577	578	71.0	578	77.7	577	44.5	619	31.8	619	31.8	623	32.1	621	27.9	618	32.0
NS4a	189	189	53.4	188	60.5	145	18.1	149	15.9	126	13.4	126	12.4	126	14.7	121	15.3
NS4b	257	257	63.3	258	60.9	257	18.3	255	15.4	255	14.6	250	18.0	252	14.6	254	17.3
NS5	889	889	81.4	889	83.9	888	60.3	905	43.7	905	43.2	905	42.7	903	42.0	898	45.0
ORF	3357	3363	70.7	3359	73.3	3341	48.1	3433	27.1	3432	27.3	3411	27.4	3414	26.9	3374	27.1

a Number of amino acid residues; ID% indicates the percentages of amino acid sequence identity. CFAV – cell fusing agent virus, CxFV – *Culex* flavivirus, CTFV – *Culex theileri* flavivirus, JEV – Japanese Encephalitis virus, MDV – Modoc virus, QBV – Quang Binh virus, TBEV – Tick-Borne Encephalitis virus, WNV – West Nile virus, YFV – Yellow Fever virus.

Protein domains were clearly identified in at least three viral proteins. The E glycoprotein (flavivirus glycoprotein superfamily-PSSM-ID 109907) evidenced a large ectodomain with at least two N-glycosylation sequences (not shown), and the putative fusion-peptide between coordinates 93 and 104. Analysis of NS3 revealed a Peptidase_S7 superfamily (PSSM-ID 144519) and a DEXDc RNA helicase domain (PSSM-ID 197446) between residues 23–145 and 194–317, respectively, with a helicase ATP-bonding domain between residues 180 and 318. Two conserved domains were detected in NS5: an S-adenosylmethionine-dependent methyltransferases superfamily domain (PSSM-ID 196296) (coordinates 4–215), and a Flavi_NS5 superfamily domain (PSSM-ID 110005) between residues 240 and 885.

Potential kinase specific (PKC) phosphorylation sites were also predicted with high probability (>0.80) in CTFV NS5 (T338, T671, S672, S836, and T856). Computer-assisted searches for nuclear localization signals (NLS) in viral proteins were negative, but the visual inspection of the C, NS3 and NS5 sequences disclosed several sections particularly rich in basic amino acid residues. These may represent potential NLS, generally obeying the consensuses suggested by Christophe et al. (2000).

### 2.5 Structural analysis of CTFV 5′- and 3′-UTR

**Fig. 4:**
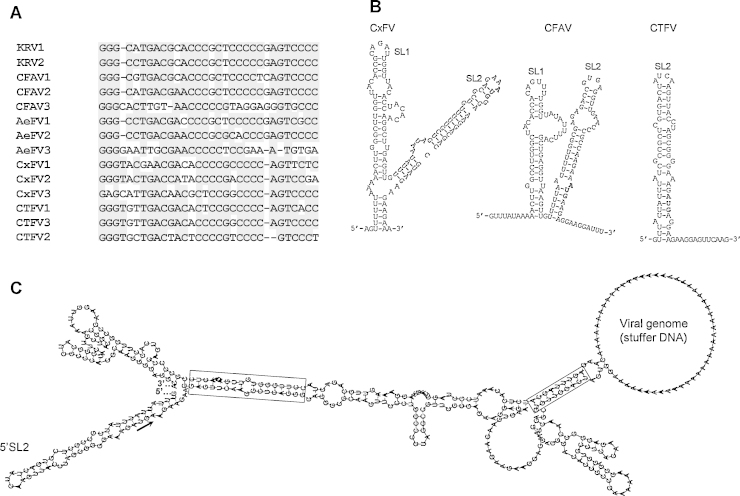
(A) Alignment of conserved GC-rich sequences located in the 3′-UTR of different insect-specific flavivirus (*Aedes* flavivirus – AeFV; cell fusing agent virus – CFAV; Kamiti River virus – KRV; *Culex* flavivirus – CxFV; *Culex theileri* flavivirus – CTFV). The most frequently found nucleotides at each position in the alignment are shaded in gray. (B) Predicted secondary structures for the *Culex* flavivirus – CxFV (AB262759), cell fusing agent virus (NC_001564) and *Culex theileri* flavivirus – CTFV (HE574574). SL indicates stem-loop structures. The AUG translation initiation codon is indicated in bold-face. (C) Computer-generated secondary structure analysis of possible interactions between the genomic plus-strand RNA ends of CTFV_178_ (HE574574). Sections of the 5′- and 3′-UTR are connected by a poly(A) insert (stuffer DNA) simulating most of the viral coding region. The AUG codon is indicated by the arrow. Regions of extended complementarity between both genome ends are boxed. The putative SL2 stem-loop that characterizes the 5′-UTR is indicated by 5′SL2.

Conserved sequence elements have been previously identified in the genomes of ISFs (Hoshino et al., 2007, Hoshino et al., 2009) in the CTFV UTR sequences, although the complete sequences of the CTFV 5′- and 3′-UTR have not been determined in full (see above). Multiple G/C-rich direct repeats with approximately 30 nt were identified at the 3′-UTR (see as CTFV1-3 in Fig. 4A). These were found to display considerable shared identity with those identified at the 3′-UTR of *Aedes*- (CFAV, KRV, AeFV) or *Culex*-associated (CxFV) flaviviruses. Our analysis suggests that they fold into a stem-loop (SL) structure (SL2, see Fig. 4B). This SL seems to be part of a more complex one forming at the viral 5′-UTR taking into account the SLs that are predicted to form at the 5′-UTR of ISFs such as CxFV and CFAV (Fig. 4B). These secondary structures seem to include the viral polyprotein AUG codon, suggesting that they might play a role in the control of initiation of translation. RNA secondary structure predictions also suggest that both ends of the CTFV genome may interact due to extensive sequence complementarity (Fig. 4C).

## 3 Discussion

The study of the flaviviruses has been stimulated by the impact that many of them have on human and animal health. Apart from the pathogenic viruses, which are biologically transmitted by hematophagous arthropods, flaviviruses also include viral agents that seem to replicate exclusively in vertebrates. A diverse group of viruses that seem to be specific to insects (ISFs) has also been tentatively assigned to the genus. Cell fusing agent virus (CFAV) was the first of these viruses to have been described (Stollar and Thomas, 1975), and its nucleotide genomic sequence was determined two decades ago (Cammisa-Parks et al., 1992). However, only recently has the study of ISFs undergone an explosion (Cook et al., 2011).

Whilst the phylogenetic relationships of ISFs with accepted flaviviruses are not clear, they have previously been suggested to form a divergent outgroup that may represent an ancestral lineage of flaviviruses (Cook and Holmes, 2006). Moreover, the identification of viral sequences integrated in the genome of mosquitoes (Crochu et al., 2004, Roiz et al., 2009, Vázquez et al., 2012), further complicates their evolutionary history. It has previously been suggested that mosquitoes carrying ISFs might be refractory to subsequent infections with pathogenic flaviviruses, as a result of superinfection exclusion (Farfan-Ale et al., 2009), but recent findings seem do not appear to support this hypothesis (Kent et al., 2010, Newman et al., 2011). In any case, ISFs seem to replicate avirulently in some insect cells *in vivo*, which is compatible with their frequent isolation, or detection of flavivirus sequence, from live mosquitoes collected all over the world (Blitvich et al., 2009, Crabtree et al., 2003, Crabtree et al., 2009, Cook et al., 2006, Cook et al., 2009, Farfan-Ale et al., 2009, Hoshino et al., 2007, Hoshino et al., 2009, Kim et al., 2009, Morales-Betoulle et al., 2008). The relative morbidity and mortality effects of ISFs on live mosquitoes and their interaction with pathogenic strains is completely unknown and hence experimental infection of colony material is a priority for future research (Cook et al., 2011).

*Culex theileri* is one of the mosquito species most frequently found in estuarine and coastal areas in the south of Portugal, especially when CO_2_-baited CDC-traps are used for the collection of adult specimens (Almeida et al., 2008, Almeida et al., 2010). As expected, during the summers of 2009 and 2010, it was found with high densities in the districts of Setúbal and Faro (Costa et al., 2011). In this report, we describe the isolation, and genetic characterization of an ISF (designated CTFV) associated with this mosquito species.

Four viral strains were initially isolated in C6/36 cells where they replicate rapidly, and accumulate in cytoplasmic cysternae, lead to the formation of cellular aggregates of various sizes, and show the size and morphology expected for a flavivirus. As expected for an ISF, they do not replicate in vertebrate (Vero) cells.

The near full-length sequences of two CTFV strains were obtained, only the 5′- and 3′-UTR remaining partially characterized. Their genome comprises an RNA molecule encoding a single ORF with a hydropathy profile, putative protease cleavage sites, and conserved protein domains similar to other flaviviruses. Notably, visual inspection of the CTFV NS5 suggests the presence of clusters of amino acids that may function as putative NLS. Although the presence of such domains in proteins encoded by viruses that, as flaviviruses, replicate in the cell cytoplasm, would, *a priori*, be unexpected, the NS5 of DENV2 and YFV (Buckley et al., 1992, Kapoor et al., 1995), the C protein of DENV2, and both the C and NS4b proteins of the Kunjin virus have previously been localized in the nucleus of infected cells (Bulich and Aaskov, 1992, Westaway et al., 1997). The identification of these proteins in the nucleus may relate to putative roles they may play in the regulation of host gene expression in virus-infected cells, as previously suggested (Hiscox, 2003). Nonetheless, it also raises doubts regarding in what extent is flavivirus replication restricted to the cell cytoplasm, especially in view of the fact that JEV, WNV and DENV-infected cells harbor active replication complexes containing phosphorylated NS5 in the nucleus (Kapoor et al., 1995, Uchil et al., 2006). Multiple putative phosphorylation sites were also observed in CTFV NS5 sequences.

Phylogenetic tree analyses placed CTFV in the ISF radiation, and in most of the trees that were constructed (the region coding for the E gene excluded) CTFV formed a monophyletic cluster with *Culex*-associated flaviviruses (Fig. 2, Fig. 3). In the region coding for the E gene tree, CTFV, QBV and CFAV sequences form a sister group with one that contains several viral CxFV (*Culex* flaviviruses) sequences isolated from *Cx. pipiens*, *Cx. quinquefasciatus* (Say), *Cx. restuans* (Theobald) *and Cx. interrogator* (Dyar & Knab, 1906). None of the phylogenetic trees, whether based on the analysis of complete ORF or specific gene sequences (E, NS3, NS5) provided any evidence for recombination involving CTFV, as recently suggested for CFAV (Cook et al., 2011).

Unlike other ISFs (Crochu et al., 2004, Roiz et al., 2009, Vázquez et al., 2012), no evidence was found for the integration of CTFV sequences in the genome of the mosquitoes from which they were isolated. However, our analysis was restricted to the attempted amplification of partial prM/E, NS3, and NS5 sequences, which does not exclude the possibility that other sections of the viral genome might be found in the DNA of *Cx. theileri*.

The 5′- and 3′-UTR of the flavivirus genomes form complex secondary structures that are conserved across the genus (Thurner et al., 2004), and that tend to hybridize leading the flavivirus genome to assume a panhandle-like structure, which has shown to be essential for viral replication (Khromykh et al., 2001). Despite the partial nature of the CTFV UTR sequences described in this report, their analyses revealed a proclivity for the formation of secondary structures, as well as for the interaction of both genome ends due to partial sequence complementarity. In particular, the 3′-UTR was shown to present multiple GC-rich conserved repeats, similar to those identified in several other ISF (Hoshino et al., 2007, Hoshino et al., 2009). It is possible that these multiple repeated sequences may be part of the 3′-UTR domains involved in interactions with host proteins, as previously documented for other flaviviruses (Lei et al., 2011, Yocupicio-Monroy et al., 2003).

The viruses described in this work have been isolated from mosquitoes collected in Portugal during the summers of 2009 and 2010, at two locations located approximately 300 km apart: Torre (district of Setúbal) and Almancil (district of Faro in the Algarve). Nevertheless, the geographical distribution of these viruses does not seem be restricted to the Portuguese territory. In fact, other viral strains with phylogenetically similar partial NS5 sequences have recently been identified by Vázquez et al. (2012) from mosquitoes collected between 2001 and 2005 in the southeast (Andalusia) and northeast (Catalonia) of Spain. This is not at all unexpected as (i) Spain and Portugal are neighbor countries in the Iberian Peninsula with no geological barriers between them, and (ii) similar *Culex*-specific ISF sequences have been detected in mosquitoes collected over a wide geographical range (Blitvich et al., 2009, Cook et al., 2009, Farfan-Ale et al., 2009, Hoshino et al., 2007, Hoshino et al., 2009, Kim et al., 2009). Furthermore, and although restricted to a very short sequence (≈160 nt; supplementary data 2), partial NS5 analysis also indicated close phylogenetic relatedness between CTFV and Wang Thong virus detected in Northern Thailand, and suggested the geographical distribution of the former (or at least that of viruses with homologous NS5 coding sequences), may extend well way from Iberia.

Curiously, while CTFV has been isolated from *Cx. theileri* mosquitoes, some of the ISF NS5 sequences identified by Vázquez et al. (2012) have been amplified from both *Cx. theileri* and *Cx. pipiens*, while Wang Thong virus was identified in *Cx. fuscocephala* mosquitoes. This suggests that CTFV (or, again, viruses with identical NS5 sequences), may not be restricted to *Cx. theileri*. Accordingly, and despite the evident segregation of *Aedes*- versus *Culex*-associated ISF in phylogenetic trees (suggesting an independent evolution of these viral lineages), Cook et al. (2011) recently found no statistical support for a host–virus co-divergence in the ISF radiation. This seems to indicate that, unlike previously suggested (Hoshino et al., 2007, Hoshino et al., 2009), ISFs may have been introduced repeatedly in different mosquito species and/or have undergone multiple host-switching potentially via horizontal transfer (Cook et al., 2011), the mechanism of which is currently not understood. In addition, the discovery of these viruses in both female and male adult mosquitoes, as well as immature forms, suggests that they are transmitted vertically (Lutomiah et al., 2007). Alternatively, as also suggested by Cook et al. (2011), the lack of statistical support for codivergence may be an artifact of undersampling of the ISFs. It remains that fact that the ISFs are likely to be hugely undersampled at present. Data regarding the host species for the “insect-specific” flaviviruses continue to be limited because for many of the publicly available “insect-specific” flavivirus sequences, molecular identification of the mosquito species and/or de-pooling to test individual specimens is not conducted, despite the fact that many *Stegomyia* and *Culex* mosquitoes exist as cryptic species complexes and/or can only be identified reliably via dissection of the male genitalia. We found that the accuracy and availability of flavivirus data for comparison with our new full ORF sequence from novel viral isolates continues to encounter difficulties due to (i) frequent lack of isolation in cell culture and/or sufficient tests to distinguish integrations of flavivirus-like sequence in mosquito genomes from viable viruses, (ii) differing taxonomic coverage across species and/or gene regions and (iii) the insufficient length of some tentative ISF sequences obtained via RT-PCR only. It remains the case that in some cases it is not proven whether the sequences are of flaviviral origin or result from integrations as some sections of apparently “insect-specific” flaviviral sequence may have been amplified from cultures in which carry-over of mosquito DNA integrations from original pool inoculum and/or from the C6/36 cell cultures themselves may have occurred. Considering the potential impact that understanding the ISFs may have on our comprehension of the emergence and maintenance of the pathogenic flaviviruses in nature, the isolation and full characterization of strains must be a future priority.

## 4 Methods

### 4.1 Mosquito collection and homogenate preparation

*Culex theileri* flaviviruses (CTFV) were isolated from pools of mosquitoes, designated 132 (*n* = 3), 153 (*n* = 50), 178 (*n* = 50), and 210 (*n* = 51) collected in July 2009 (178) and 2010 (132, 153, and 210) with CDC traps baited with CO_2_. Mosquito collections were carried out in (i) a rural area surrounded by rice fields and salt marshes (Torre/Comporta – 38°21′5.3″N, 8°47′11.7″W) and located approximately 20 km south of the city of Setúbal (pool 153), (ii) in a rural area (Gâmbia – 38°33′10.6″N, 8°45′35.9″W) bordered by salt marshes located about 15 km east of Setúbal (pool 132), (iii) close to a seaside lagoon and an urban waste water treatment plant (Dunas Douradas) in the Algarve (pool 178), near Almancil (district of Faro, coordinates 37°2′42.6″N, 8°3′8.4″W), and (iv) in the vicinity of a nature reserve located close to the Portuguese/Spanish border (pool 210) in the Algarve, also in the district of Faro (37°12′32.6″N, 7°27′51.0″W). All captured mosquitoes were identified using Ribeiro and Ramos (1999) identification keys, considering as recognized European *taxa* those referred by Ramsdale and Snow (1999).

Mosquito homogenates were prepared by mechanical homogenization of adult mosquitoes using glass beads (Huang et al., 2001). Homogenates were cleared by centrifugation at 13,000 × *g* (4 °C for 10 min), sterilized through 0.22 μm disposable PVDF filters (Millex-GV, Millipore Corp., Bedford, USA), and kept at −80 °C.

### 4.2 Cell culture and virus isolation

The *Stegomyia albopicta* C6/36 cell line was used for virus isolation. Cells were maintained at 28 °C (in the absence of CO_2_) in L-15 Leibovitz Medium (Lonza, Walkersville/MD, USA) supplemented with 10% heat inactivated fetal bovine serum (FBS) (Lonza, Walkersville/MD, USA), 2 mM l-glutamine (Gibco BRL, Gaitherburg/MD, USA), 100 U/ml penicillin and 100 μg/ml streptomycin (Gibco BRL, Gaitherburg/MD, USA) and 1× triptose phosphate broth (AppliChem GmbH, Darmstadt, Germany). Viral replication in vertebrate cells was tested using the Vero E6 cell line (ATCC CRL-1586) kept at 37 °C with 5% CO_2_ in Dulbecco's Modified Eagle Medium (Lonza, Walkersville/MD, USA) supplemented with 10% FBS.

Approximately 500 μl of filter-sterilized mosquito homogenate was diluted in an equal volume of phosphate buffered saline (PBS) and inoculated onto semi-confluent layers of C6/36 cells grown in T25 culture flasks (Nunc, Roskilde, Denmark). After 1 h at room temperature, the viral inoculum was removed, 5 ml of L-15 Leibovitz Medium (5% FBS) was added to each flask, and the cell cultures were incubated at 28 °C for a week. Culture supernatants collected after the third blind passage were used as viral stocks, and stored at −80 °C. Cytopathic effect (CPE) was determined by microscopic observation of the inoculated cell cultures.

### 4.3 Transmission electron microscopy (TEM)

C6/36 cell cultures were infected with 1 ml of viral stocks (only the 153 isolate was used). When CPE became evident (48 h post-infection) the C6/36 cells were scraped from the culture flask and prepared for TEM examination. Briefly, infected cells were fixed sequentially in 3% glutaraldehyde (in cacodylate buffer), osmium tetroxide (in the same buffer) and uranyl acetate (in bi-distilled water). Dehydration was carried out in increasing concentrations of ethanol. After passage through propylene oxide, the samples were embedded in Epon-Araldite, using SPI-Pon as an Epon 812 substitute. Thin sections were made with glass or diamond knives and stained with 2% aqueous uranyl acetate and Reynold's lead citrate. The stained sections were studied and photographed in a JEOL 100-SX electron microscope.

### 4.4 Nucleotide sequence amplification and DNA sequencing

Viral RNA was extracted from 150 μl of culture supernatant using the ZR Viral RNA Kit™ (Zymo Research, Irvine, CA) according to manufacturer's recommendations. Total RNA was also extracted from C6/36 infected and non-infected cells using the INSTANT Virus RNA kit (Analytik Jena AG, Jena, Germany), also following the supplier's instructions. Reverse transcription of viral RNA was carried out with the RevertAid™ H Minus First Strand cDNA Synthesis kit and random hexaprimers (Fermentas, Vilnius, Lithuania), using 5 μl of the RNA extract. The obtained cDNA served as template for the amplification of viral sequences using Phusion™ High Fidelity DNA Polymerase (Finnzymes, Vantaa, Finland), and the oligonucleotides listed in supplementary Table 1. These were complementary to the most conserved regions displayed in multiple sequence alignments produced with MAFFT vs. 6 (Katoh and Toh, 2008), of 7 reference Culex flavivirus sequences deposited in the GenBank public database under accession numbers AB377213 (strain NIID-21-2), EU879060 (strain CxFV-Mex07), FJ502995 (strain HOU24518), FJ644291 (strain VN180), FJ663034 (strain Iowa07), GQ165808 (strain Uganda08), NC_008604 (strain Tokyo).

DNA amplicons were purified with the DNA Clean & Concentrator™-5 (Zymo Research, Irvine, CA) and either directly sequenced or cloned in either pGEM^®^-T Easy (Promega, Madison, WI) or CloneJET™ (Fermentas, Vilnius, Lithuania) using *Escherichia coli* NovaBlue (Merck KGaA, Darmstadt, Germany) as host, prior to DNA sequencing.

Partial mitochondrial cytochrome c oxidase subunit I (COI) sequences were amplified from total genomic DNA, extracted from mosquito homogenates with the ZymoBead™ Genomic DNA kit (Zymo Research, Irvine, CA), and the PuRe Taq Ready-to-Go PCR Beads (GE Healthcare, Dornstadt, Germany), using primers and reaction conditions previously described (Cook et al., 2009; described by Folmer et al., 1994). The obtained amplicons were purified, cloned in pGEM^®^T Easy (Promega, Madison, WI), and sequenced.

### 4.5 Nucleotide and amino acid sequence analyses

The near full-length genomic sequences of CTFV were assembled using the CAP Contig Manager tool available in the BioEdit 7.0.2. software (Hall, 1999). Nucleotide and protein similarity searches were carried out through the NCBI web server using BLASTn and BLASTx (http://blast.ncbi.nlm.nih.gov/Blast.cgi).

Phylogenetic relationships were inferred from nucleotide sequences aligned with MAFFT vs. 6 (care was taken so as to maintain codon alignment), using the evolutionary model indicated by jModeltest (Posada, 2008), and defined with Akaike information criterion (GTR+I+Γ; used for Bayesian analysis). Trees were constructed using MrBayes v3.0b4 (Ronquist and Huelsenbeck, 2003) or MEGA5.01 (Tamura et al., 2011; used for Neighbor-Joining (NJ) analysis). The Bayesian analyses consisted of 10 × 10^6^ generations starting from a random tree and four Markov chains with default heating values sampled every 100th generation. The first 10% sampled trees were discarded (burn-in). To prevent reaching only apparent stationarity, two separate runs were conducted for each analysis.

For the analysis of partial NS5 nt sequences, Bayesian or Neighbor-Joining (NJ) trees were constructed (in the latter case from genetic distance matrixes corrected with the Kimura 2-parameter (K2P) formula). The reliability of the inferred NJ trees was evaluated by bootstrap analysis of or 1000 data replicates. The final trees were manipulated for display using FigTree v.1.2.2 (available at http://tree.bio.ed.ac.uk/software/figtree/).

Phylogenetic relationships were also inferred from amino acid sequence alignments produced with MUSCLE (Edgar, 2004), and using a Bayesian approach. The alignments were treated with GBlocks (Castresana, 2000) to remove highly variable regions of the alignment where homology was dubious.

Mosquito taxonomic classification based on a molecular approach was carried out by BLAST searches and phylogenetic analysis of the obtained COI sequences using BOLD-ID (Barcode of Life Data System Identification engine) available at www.boldsystem.org/view/login.php.

Genetic diversity between protein sequences was calculated using MEGA5.01 (corrected with Poisson model) from alignments obtained using MAFFT. Protein hydropathy plots were constructed with the Gene Runner 3.05 software (available for download at http://www.generunner.net/) using the Kyte–Doolittle hydropathy scale and a window of 10 amino acid residues. Protein motifs were identified by running Pfam and Prosite protein profile (using the Motif Search tool: http://www.genome.jp/tools/motif/) and conserved domain searches (using the Web CD-search tool: http://www.ncbi.nlm.nih.gov/Structure/bwrpsb/bwrpsb.cgi). Potential nuclear localization signals were tentatively identified with PredictProtein (available at www.predictprotein.org) and by visual inspection of protein sequences using the consensus defined by Christophe et al. (2000) as a reference. Transmembrane helices in protein sequences were predicted with SOSUI (http://bp.nuap.nagoya-u.ac.jp/sosui/) and TMHMM Server v. 2.0 (available at http://www.cbs.dtu.dk/services/TMHMM/). Access to the latter server also provided tools for the analysis of potential N-glycosylation sequences (NetNGlyco) and kinase specific eukaryotic protein phosphoylation sites (NetPhosK 1.0).

### 4.6 Analysis of the 5′- and 3′-UTR and cyclization of the CTFV RNA

Analyses of 3′-UTR focused on the visual identification of conserved and repeated sequence motifs, as well as direct and inverted repeats, commonly found in the genome of flaviviruses.

Folding patterns of the 5′- and 3′-UTR were predicted using default parameters of online RNA folding services provided by the MFOLD (http://mfold.rna.albany.edu/?q=mfold) and RNAfold (http://rna.tbi.univie.ac.at/cgi-bin/RNAfold.cgi) web servers. The multiple structures predicted by both servers were compared to those obtained for the 5′- and 3′-UTR of *Culex* flavivirus (CxFv strain Tokyo; NC_008604), and cell fusing agent virus (NC_001564). To investigate the possible cyclization of the CTFV RNA, partial 5′- and 3′-UTR sequences were artificially linked by a flexible string of multiple adenine nucleotides (stuffer DNA), and the RNA folding patterns were predicted by MFOLD and RNAfold.

### 4.7 Nucleotide sequence accession numbers

The nucleotide sequences of the CTFV near full-length genomes reported in this work have been deposited in the GenBank/EMBL/DDBJ databases under accession numbers HE574573 (isolate 153) and HE574574 (isolate 178). The five partial CTFV NS5 sequences reported in this work have been assigned the accession numbers FR873476–FR873480FR873476FR873477FR873478FR873479FR873480. Partial *Cx. theileri* COI sequences were deposited in the GenBank/EMBL/DDBJ database under accession numbers HE610457–HE610460HE610457HE610458HE610459HE610460.

## Appendix A Supplementary data

Supplementary data 1 Molecular analysis of COI sequences amplified from the pools of mosquitoes from which CTFV strains was isolated. The phylogenetic trees (Neighbor-Joining) were constructed using genetic distances corrected with the Kimura 2-parameter formula, based on multiple alignments of nucleotide sequences (all codon positions were used). The scale indicates 0.2% of genetic diversity. The COI sequences from amplicons obtained from the 132, 153, 178, and 210 pools were unambiguously identified (100% probability) as *Culex theileri*. All the trees showed similar topologies. The example given indicates the analysis of the COI sequences from pool 153 (indicated by the arrow, at the bottom).

Supplementary data 2 Bayesian analysis of multiply aligned partial NS5 sequences (153 unambiguously aligned nucleotides; covering positions 8918–9076 relative to CxFV_NC_008604_) from different flaviviruses indicated by virus name and accession number (for some, the strain name is also indicated in parentheses). The numbers at specific branches indicate significant posterior probability values (>0.80). The scale bar indicates 20% of genetic distance.

Supplementary data 3 Neighbor-Joining tree based on the analysis of genetic distances calculated (with the Kimura 2-parameter formula) from multiple alignments of partial NS5 sequences from different flaviviruses indicated by virus name and accession number (for some, the strain name is also indicated in parentheses). The numbers at specific branches indicate the bootstrap values (>75%) resulting from 1000 resamplings of the original sequence dataset. The monophyletic cluster including CTFV sequences, limited by a dotted box, showed a K2P-corrected genetic diversity (*d*) of 1%. One sequence obtained from strains 132, 178 and 210. The amplicon obtained from the 153 strain was cloned in pGEM^®^-T Easy, and two independent clones were analyzed (Clone1 and Clone2). The scale bar indicates 10% of genetic distance.

Supplementary data 4 Bayesian phylogenetic analysis of flavivirus ORF nucleotide sequences. Posterior probability values ≥0.95 are indicated at specific branches. The CTFV sequences are indicated by the arrows. The scale bar indicates 15% of genetic diversity.

Supplementary data 5 Bayesian phylogenetic analysis of flavivirus E protein sequences. Posterior probability values ≥0.95 are indicated at specific branches. The CTFV sequences are indicated by the strain designation (153 and 178; indicated by an arrow). The scale bar indicates 20% of genetic diversity.
