# Supplementary figures and images for: Genetic characterization of an insect-specific flavivirus isolated from Culex theileri mosquitoes collected in southern Portugal
Source: Virus Res. 2012 Aug;167(2):152–61. doi: 10.1016/j.virusres.2012.04.010 (PMC3919203; doi:10.1016/j.virusres.2012.04.010)

## Slide 1
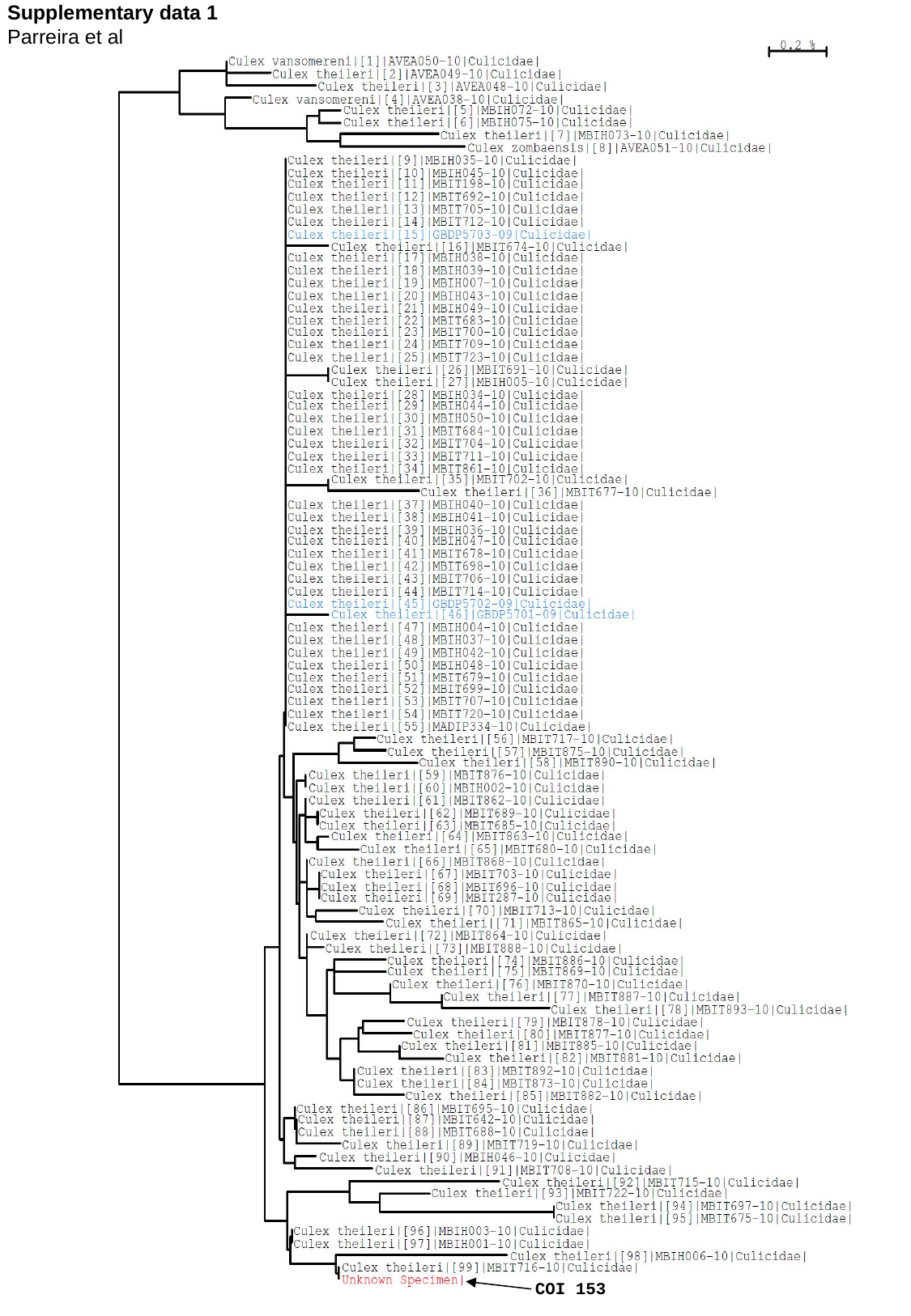

Supplementary data 1
Parreira et al
COI 153

Supplement: Supplementary data 1 — Molecular analysis of COI sequences amplified from the pools of mosquitoes from which CTFV strains was isolated. The phylogenetic trees (Neighbor-Joining) were constructed using genetic distances corrected with the Kimura 2-parameter formula, based on multiple alignments of nucleotide sequences (all codon positions were used). The scale indicates 0.2% of genetic diversity. The COI sequences from amplicons obtained from the 132, 153, 178, and 210 pools were unambiguously identified (100% probability) as Culex theileri. All the trees showed similar topologies. The example given indicates the analysis of the COI sequences from pool 153 (indicated by the arrow, at the bottom). [file mmc1.ppt]

## Slide 1
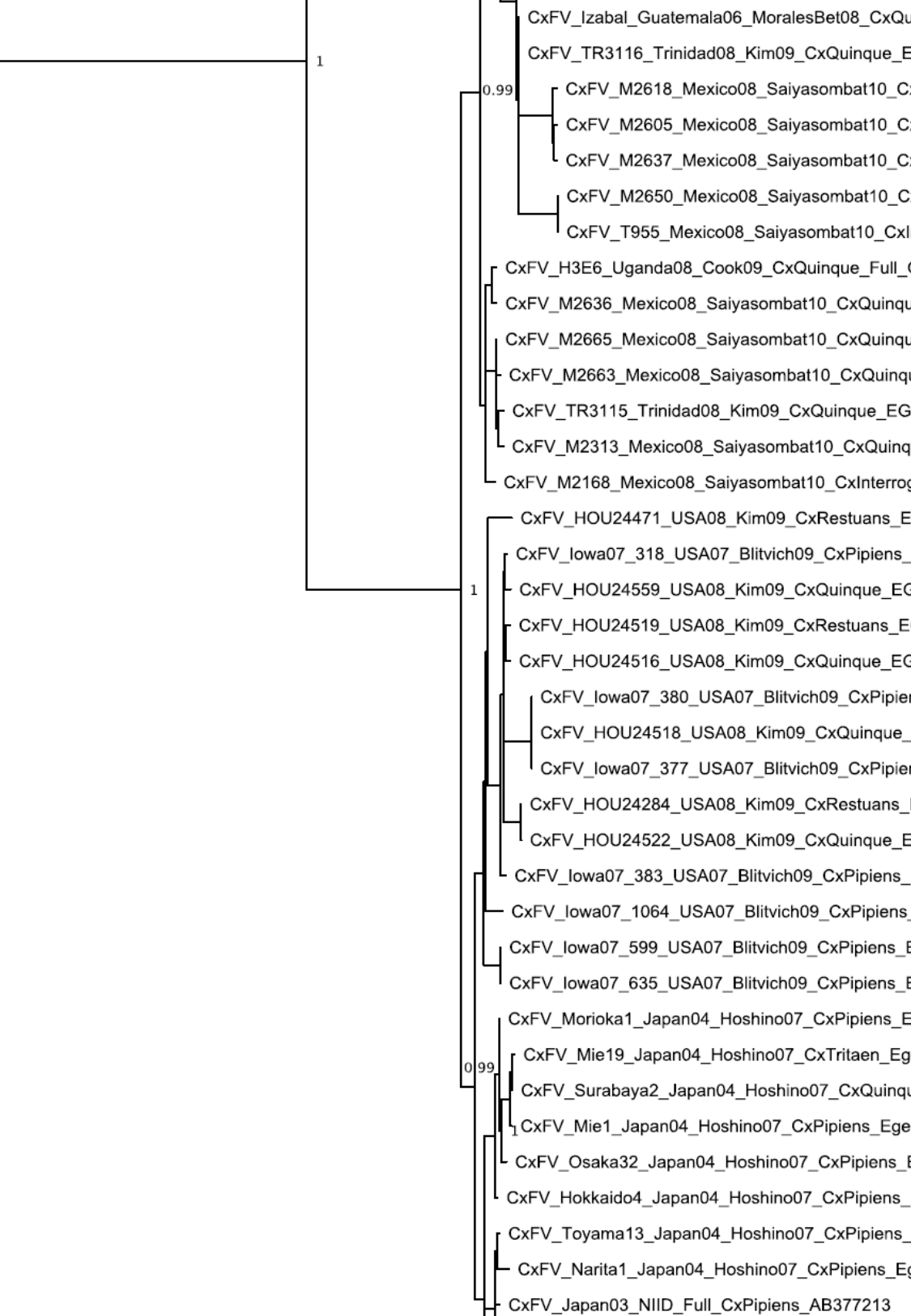

Supplementary data 5
Parreira et al
CTFV178
CTFV153

Supplement: Supplementary data 5 — Bayesian phylogenetic analysis of flavivirus E protein sequences. Posterior probability values ≥0.95 are indicated at specific branches. The CTFV sequences are indicated by the strain designation (153 and 178; indicated by an arrow). The scale bar indicates 20% of genetic diversity. [file mmc5.ppt]
