# Supplementary material for: Genetic characterization of an insect-specific flavivirus isolated from Culex theileri mosquitoes collected in southern Portugal
Source: Virus Res. 2012 Aug;167(2):152–61. doi: 10.1016/j.virusres.2012.04.010 (PMC3919203; doi:10.1016/j.virusres.2012.04.010)
Supplement: Supplementary data 2 — Bayesian analysis of multiply aligned partial NS5 sequences (153 unambiguously aligned nucleotides; covering positions 8918–9076 relative to CxFVNC_008604) from different flaviviruses indicated by virus name and accession number (for some, the strain name is also indicated in parentheses). The numbers at specific branches indicate significant posterior probability values (>0.80). The scale bar indicates 20% of genetic distance. [file mmc2.ppt]

## Slide 1
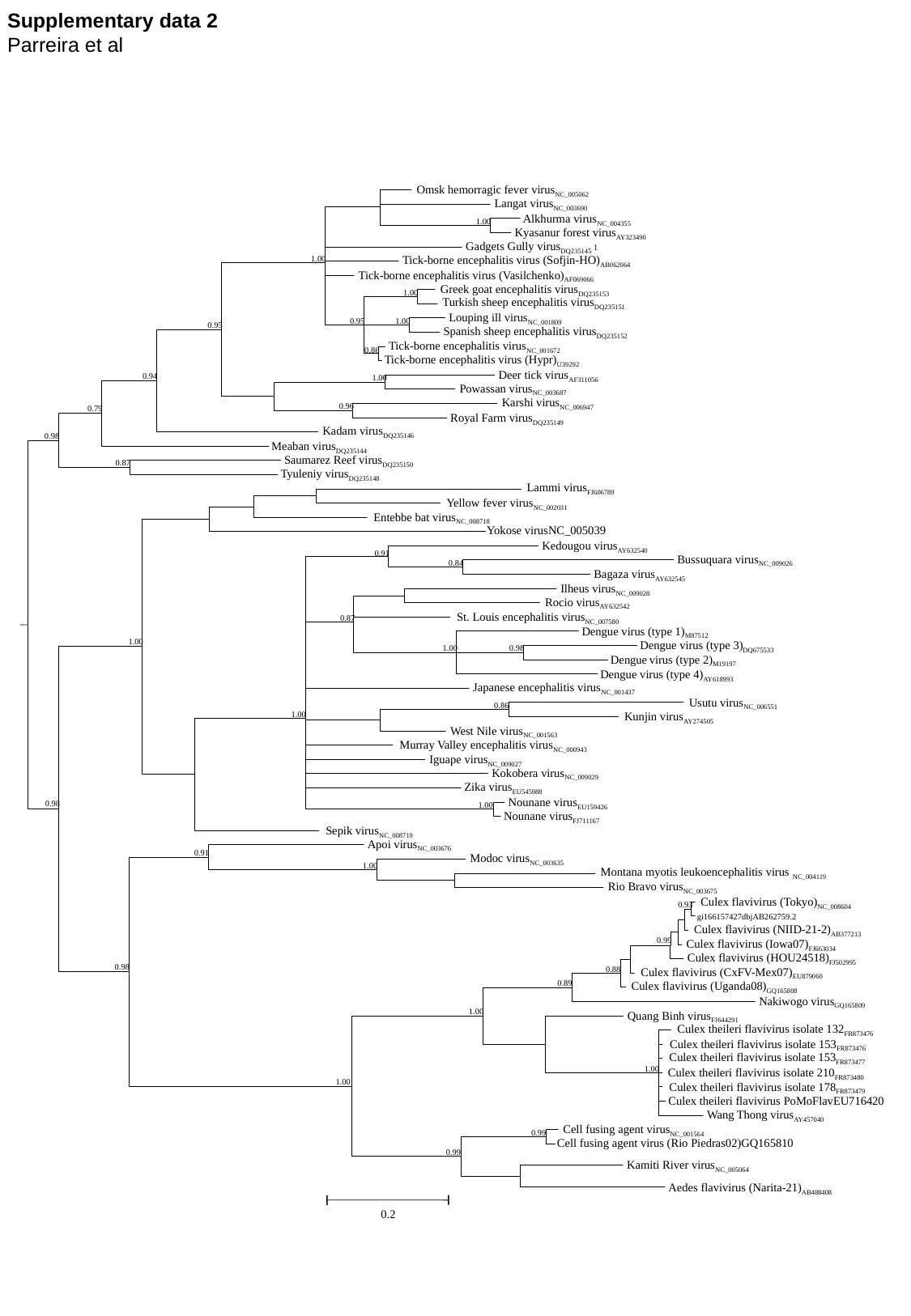

Supplementary data 2
Parreira et al
Omsk hemorragic fever virusNC_005062
Langat virusNC_003690
Alkhurma virusNC_004355
1.00
Kyasanur forest virusAY323490
Gadgets Gully virusDQ235145 1
Tick-borne encephalitis virus (Sofjin-HO)AB062064
1.00
Tick-borne encephalitis virus (Vasilchenko)AF069066
Greek goat encephalitis virusDQ235153
1.00
Turkish sheep encephalitis virusDQ235151
Louping ill virusNC_001809
0.95
1.00
0.95
Spanish sheep encephalitis virusDQ235152
Tick-borne encephalitis virusNC_001672
0.86
Tick-borne encephalitis virus (Hypr)U39292
Deer tick virusAF311056
0.94
1.00
Powassan virusNC_003687
Karshi virusNC_006947
0.96
0.79
Royal Farm virusDQ235149
Kadam virusDQ235146
0.98
Meaban virusDQ235144
Saumarez Reef virusDQ235150
0.87
Tyuleniy virusDQ235148
Lammi virusFJ606789
Yellow fever virusNC_002031
Entebbe bat virusNC_008718
Yokose virusNC_005039
Kedougou virusAY632540
0.91
Bussuquara virusNC_009026
0.84
Bagaza virusAY632545
Ilheus virusNC_009028
Rocio virusAY632542
St. Louis encephalitis virusNC_007580
0.87
Dengue virus (type 1)M87512
1.00
Dengue virus (type 3)DQ675533
1.00
0.98
Dengue virus (type 2)M19197
Dengue virus (type 4)AY618993
Japanese encephalitis virusNC_001437
Usutu virusNC_006551
0.86
1.00
Kunjin virusAY274505
West Nile virusNC_001563
Murray Valley encephalitis virusNC_000943
Iguape virusNC_009027
Kokobera virusNC_009029
Zika virusEU545988
Nounane virusEU159426
0.98
1.00
Nounane virusFJ711167
Sepik virusNC_008719
Apoi virusNC_003676
0.91
Modoc virusNC_003635
1.00
Montana myotis leukoencephalitis virus NC_004119
Rio Bravo virusNC_003675
Culex flavivirus (Tokyo)NC_008604
0.93
gi166157427dbjAB262759.2
Culex flavivirus (NIID-21-2)AB377213
0.99
Culex flavivirus (Iowa07)FJ663034
Culex flavivirus (HOU24518)FJ502995
0.98
0.88
Culex flavivirus (CxFV-Mex07)EU879060
0.89
Culex flavivirus (Uganda08)GQ165808
Nakiwogo virusGQ165809
1.00
Quang Binh virusFJ644291
Culex theileri flavivirus isolate 132FR873476
Culex theileri flavivirus isolate 153FR873476
Culex theileri flavivirus isolate 153FR873477
1.00
Culex theileri flavivirus isolate 210FR873480
1.00
Culex theileri flavivirus isolate 178FR873479
Culex theileri flavivirus PoMoFlavEU716420
Wang Thong virusAY457040
Cell fusing agent virusNC_001564
0.99
Cell fusing agent virus (Rio Piedras02)GQ165810
0.99
Kamiti River virusNC_005064
Aedes flavivirus (Narita-21)AB488408
0.2
