# Supplementary material for: Genetic characterization of an insect-specific flavivirus isolated from Culex theileri mosquitoes collected in southern Portugal
Source: Virus Res. 2012 Aug;167(2):152–61. doi: 10.1016/j.virusres.2012.04.010 (PMC3919203; doi:10.1016/j.virusres.2012.04.010)
Supplement: Supplementary data 3 — Neighbor-Joining tree based on the analysis of genetic distances calculated (with the Kimura 2-parameter formula) from multiple alignments of partial NS5 sequences from different flaviviruses indicated by virus name and accession number (for some, the strain name is also indicated in parentheses). The numbers at specific branches indicate the bootstrap values (>75%) resulting from 1000 resamplings of the original sequence dataset. The monophyletic cluster including CTFV sequences, limited by a dotted box, showed a K2P-corrected genetic diversity (d) of 1%. One sequence obtained from strains 132, 178 and 210. The amplicon obtained from the 153 strain was cloned in pGEM®-T Easy, and two independent clones were analyzed (Clone1 and Clone2). The scale bar indicates 10% of genetic distance. [file mmc3.ppt]

## Slide 1
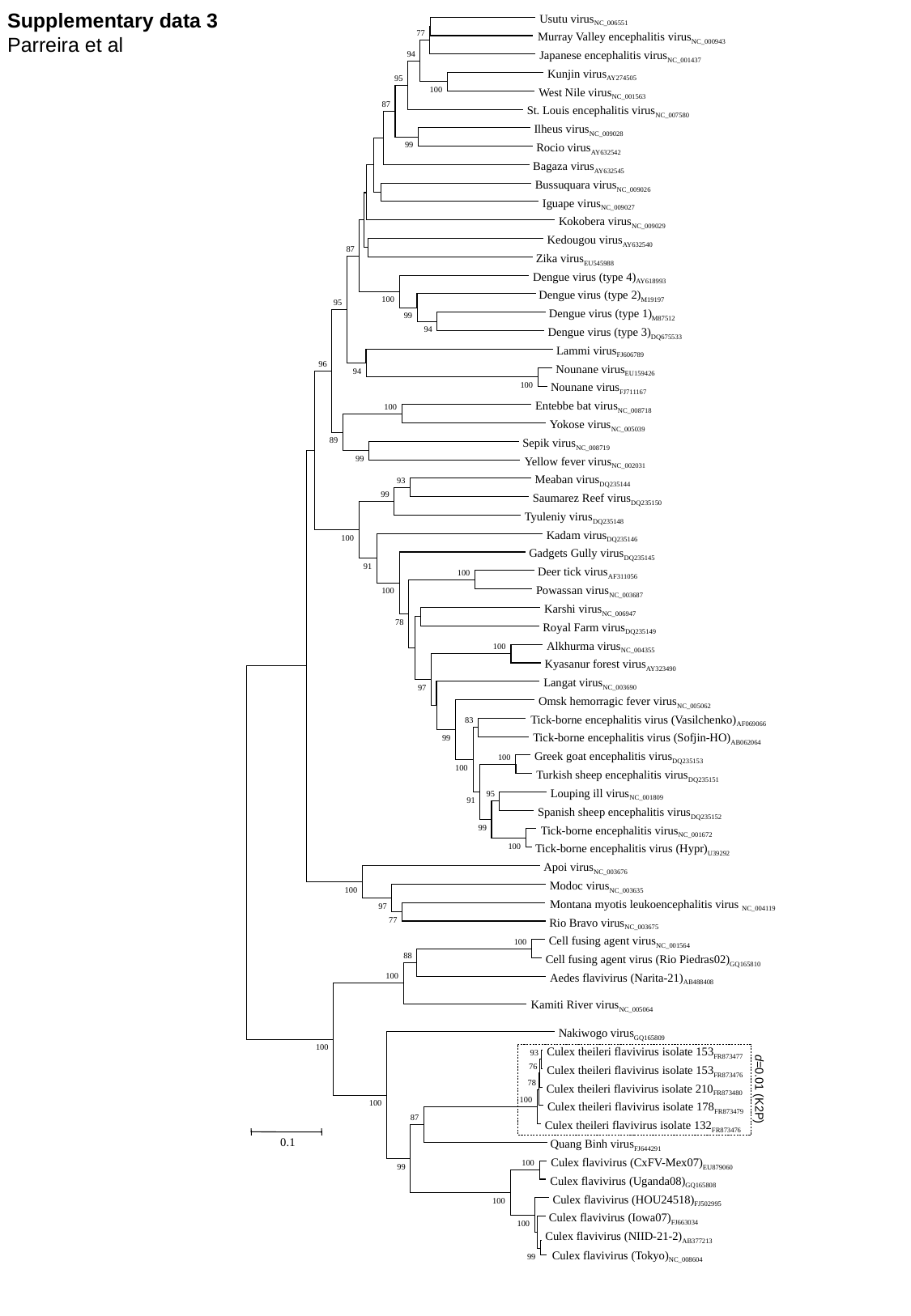

Supplementary data 3
Parreira et al
Usutu virusNC_006551
77
Murray Valley encephalitis virusNC_000943
Japanese encephalitis virusNC_001437
94
Kunjin virusAY274505
95
100
West Nile virusNC_001563
87
St. Louis encephalitis virusNC_007580
Ilheus virusNC_009028
99
Rocio virusAY632542
Bagaza virusAY632545
Bussuquara virusNC_009026
Iguape virusNC_009027
Kokobera virusNC_009029
Kedougou virusAY632540
87
Zika virusEU545988
Dengue virus (type 4)AY618993
Dengue virus (type 2)M19197
100
95
Dengue virus (type 1)M87512
99
94
Dengue virus (type 3)DQ675533
Lammi virusFJ606789
96
Nounane virusEU159426
94
100
Nounane virusFJ711167
Entebbe bat virusNC_008718
100
Yokose virusNC_005039
89
Sepik virusNC_008719
99
Yellow fever virusNC_002031
Meaban virusDQ235144
93
99
Saumarez Reef virusDQ235150
Tyuleniy virusDQ235148
Kadam virusDQ235146
100
Gadgets Gully virusDQ235145
91
Deer tick virusAF311056
100
Powassan virusNC_003687
100
Karshi virusNC_006947
78
Royal Farm virusDQ235149
Alkhurma virusNC_004355
100
Kyasanur forest virusAY323490
Langat virusNC_003690
97
Omsk hemorragic fever virusNC_005062
Tick-borne encephalitis virus (Vasilchenko)AF069066
83
Tick-borne encephalitis virus (Sofjin-HO)AB062064
99
Greek goat encephalitis virusDQ235153
100
100
Turkish sheep encephalitis virusDQ235151
Louping ill virusNC_001809
95
91
Spanish sheep encephalitis virusDQ235152
99
Tick-borne encephalitis virusNC_001672
100
Tick-borne encephalitis virus (Hypr)U39292
Apoi virusNC_003676
Modoc virusNC_003635
100
Montana myotis leukoencephalitis virus NC_004119
97
77
Rio Bravo virusNC_003675
Cell fusing agent virusNC_001564
100
88
Cell fusing agent virus (Rio Piedras02)GQ165810
Aedes flavivirus (Narita-21)AB488408
100
Kamiti River virusNC_005064
Nakiwogo virusGQ165809
100
Culex theileri flavivirus isolate 153FR873477
93
76
Culex theileri flavivirus isolate 153FR873476
d=0.01 (K2P)
78
Culex theileri flavivirus isolate 210FR873480
100
100
Culex theileri flavivirus isolate 178FR873479
87
Culex theileri flavivirus isolate 132FR873476
0.1
Quang Binh virusFJ644291
Culex flavivirus (CxFV-Mex07)EU879060
100
99
Culex flavivirus (Uganda08)GQ165808
Culex flavivirus (HOU24518)FJ502995
100
Culex flavivirus (Iowa07)FJ663034
100
Culex flavivirus (NIID-21-2)AB377213
Culex flavivirus (Tokyo)NC_008604
99
