# Supplementary material for: Genetic characterization of an insect-specific flavivirus isolated from Culex theileri mosquitoes collected in southern Portugal
Source: Virus Res. 2012 Aug;167(2):152–61. doi: 10.1016/j.virusres.2012.04.010 (PMC3919203; doi:10.1016/j.virusres.2012.04.010)
Supplement: Supplementary data 4 — Bayesian phylogenetic analysis of flavivirus ORF nucleotide sequences. Posterior probability values ≥0.95 are indicated at specific branches. The CTFV sequences are indicated by the arrows. The scale bar indicates 15% of genetic diversity. [file mmc4.ppt]

## Slide 1
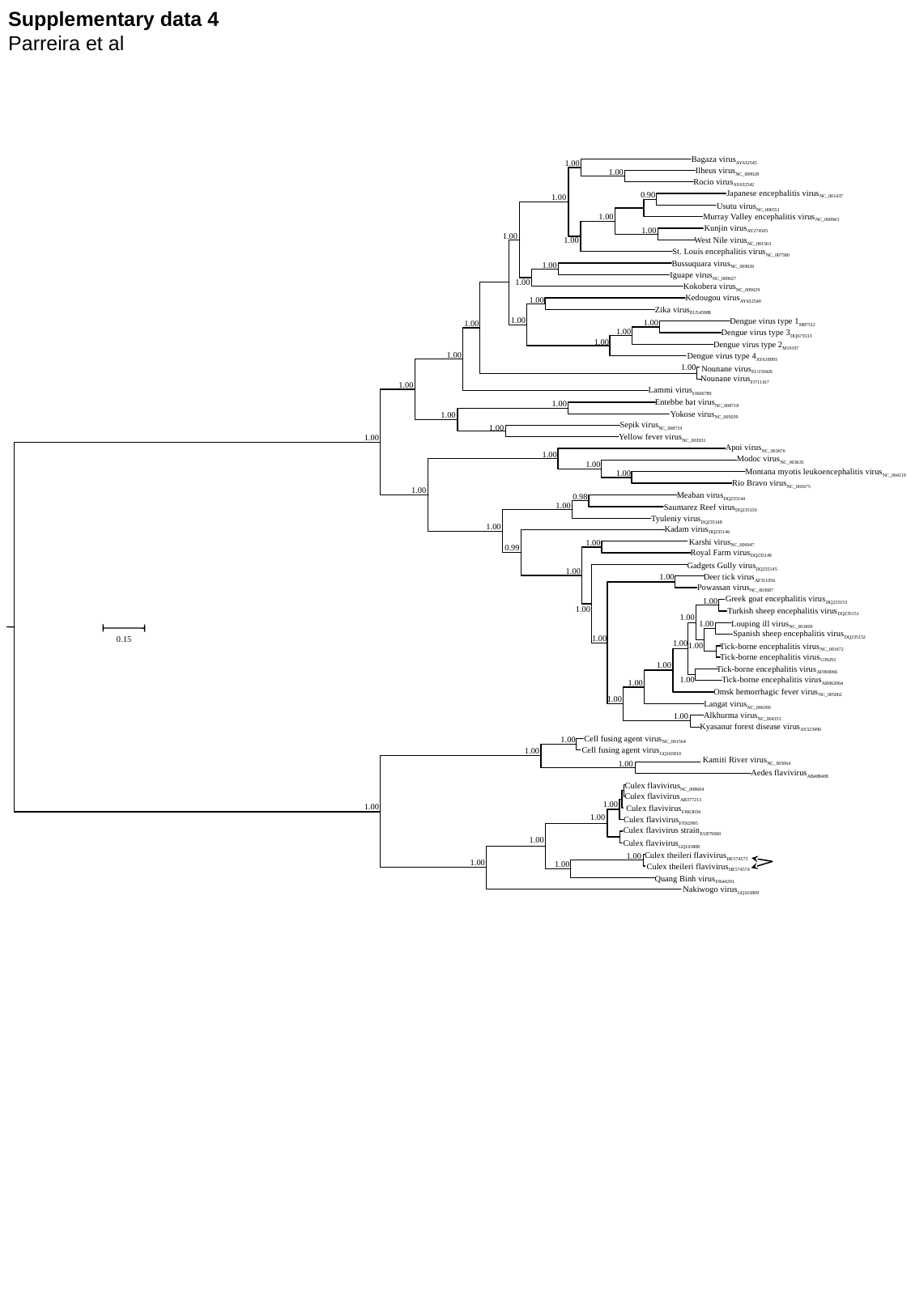

Supplementary data 4
Parreira et al
Bagaza virusAY632545
1.00
Ilheus virusNC_009028
1.00
Rocio virusAY632542
Japanese encephalitis virusNC_001437
0.90
1.00
Usutu virusNC_006551
Murray Valley encephalitis virusNC_000943
1.00
Kunjin virusAY274505
1.00
1.00
1.00
West Nile virusNC_001563
St. Louis encephalitis virusNC_007580
Bussuquara virusNC_009026
1.00
Iguape virusNC_009027
1.00
Kokobera virusNC_009029
Kedougou virusAY632540
1.00
Zika virusEU545988
1.00
Dengue virus type 1M87512
1.00
1.00
1.00
Dengue virus type 3DQ675533
1.00
Dengue virus type 2M19197
1.00
Dengue virus type 4AY618993
1.00
Nounane virusEU159426
Nounane virusFJ711167
1.00
Lammi virusFJ606789
Entebbe bat virusNC_008718
1.00
Yokose virusNC_005039
1.00
Sepik virusNC_008719
1.00
Yellow fever virusNC_002031
1.00
Apoi virusNC_003676
1.00
Modoc virusNC_003635
1.00
Montana myotis leukoencephalitis virusNC_004119
1.00
Rio Bravo virusNC_003675
1.00
Meaban virusDQ235144
0.98
1.00
Saumarez Reef virusDQ235150
Tyuleniy virusDQ235148
1.00
Kadam virusDQ235146
Karshi virusNC_006947
1.00
0.99
Royal Farm virusDQ235149
Gadgets Gully virusDQ235145
1.00
1.00
Deer tick virusAF311056
Powassan virusNC_003687
Greek goat encephalitis virusDQ235153
1.00
1.00
Turkish sheep encephalitis virusDQ235151
1.00
1.00
Louping ill virusNC_001809
Spanish sheep encephalitis virusDQ235152
1.00
0.15
1.00
1.00
Tick-borne encephalitis virusNC_001672
Tick-borne encephalitis virusU39292
1.00
Tick-borne encephalitis virusAF069066
1.00
Tick-borne encephalitis virusAB062064
1.00
Omsk hemorrhagic fever virusNC_005062
1.00
Langat virusNC_006390
Alkhurma virusNC_004355
1.00
Kyasanur forest disease virusAY323490
Cell fusing agent virusNC_001564
1.00
Cell fusing agent virusGQ165810
1.00
Kamiti River virusNC_005064
1.00
Aedes flavivirusAB488408
Culex flavivirusNC_008604
Culex flavivirusAB377213
1.00
1.00
Culex flavivirusFJ663034
1.00
Culex flavivirusFJ502995
Culex flavivirus strainEU879060
1.00
Culex flavivirusGQ165808
Culex theileri flavivirusHE574573
1.00
1.00
1.00
Culex theileri flavivirusHE574574
Quang Binh virusFJ644291
Nakiwogo virusGQ165809
