# Supplementary material for: Genetic characterization of an insect-specific flavivirus isolated from Culex theileri mosquitoes collected in southern Portugal
Source: Virus Res. 2012 Aug;167(2):152–61. doi: 10.1016/j.virusres.2012.04.010 (PMC3919203; doi:10.1016/j.virusres.2012.04.010)
Supplement: Supplementary file 6 [file mmc6.doc]

Supplementary Table 1 – Primers used for synthesis and amplification of CTFV cDNA.

| **Primer designation** | **Position^1^** | **Orientation** | **Sequence (5’-3’)** |
| --- | --- | --- | --- |
| F16 | 1-27 | Forward | AGTTTTTAAAAACTTYRGCTTGGTTAM |
| SeqF | 719-739 | Forward | CGCTACCATCGCTGTCTTCCG |
| SeqG | 1647-1667 | Reverse | GTGTAGCGATCGCGTCATCCC |
| F13 | 2219-2241 | Forward | GATGTGGGTTGCGGATTTGACCC |
| F17 | 2524-2547 | Reverse | CCGATCTTGACTCCATGCACAGCC |
| F10 | 3570-3596 | Forward | GGTCTTGGATTGGATTGTCGTACTCGC |
| F12 | 3850-3873 | Reverse | AGTACTACTGTGTCCACAGGCACG |
| SeqC | 5011-5031 | Forward | GTTCGTTGACTGGCATCCAGG |
| F11 | 5212-5239 | Reverse | CGTTAGCGTCGCGTGGCAAGCCACCGTG |
| F6 | 5732-5754 | Forward | ATGCTGGAGAARGTTGGCATCAC |
| SeqD | 5856-5879 | Reverse | TCTGTGCCTCAGACCAACAAGCCC |
| F4 | 7712-7738 | Forward | GAYYTHGGNTGYGGYMGVGGNGGMTGG |
| F7 | 7892-7917 | Reverse | ATGTCACAYAYGATSGTGTTGCAATC |
| Flavi1* | 8831-8850 | Forward | TGYRTNTAYAACAYVATGGG |
| F14 | 8895-8922 | Forward | CACGSATYATCTGGTACATGTGGCTSGG |
| Flavi3* | 8903-8922 | Forward | ATHTGGTWYATGTGGYTNGG |
| Flavi2* | 9081-9100 | Reverse | GTGTCCCANCCDGCDRYRTC |
| SeqE | 9630-9651 | Forward | GTGATGGACGGGAGATTATCGC |
| F15 | 10703-10723 | Reverse | ATCCTGTGCGCCCTCACCTTG |

^1^ The coordinates indicated refer to those of the *Culex* flavivirus genome (CxFV; NC_008604).
